# Insulin-regulated aminopeptidase contributes to setting the intensity of FcR-mediated inflammation

**DOI:** 10.3389/fimmu.2022.1029759

**Published:** 2022-10-27

**Authors:** Manuela Bratti, Shamila Vibhushan, Cyril Longé, Despoina Koumantou, Gaël Ménasché, Marc Benhamou, Nadine Varin-Blank, Ulrich Blank, Loredana Saveanu, Sanae Ben Mkaddem

**Affiliations:** ^1^ Université Paris Cité, Centre de Recherche sur l’Inflammation, Institut National de la Santé et de la Recherche Médicale (INSERM) Unité Mixte de Recherche (UMR)1149, Centre National de la Recherche Scientifique (CNRS) Equipe Mixte de Recherche(EMR)-8252, Faculté de Médecine site Bichat, Paris, France; ^2^ Université Paris Cité, Laboratoire d’Excellence INFLAMEX, Paris, France; ^3^ Université Paris Cité, Imagine Institute, Laboratory of Molecular basis of altered immune homeostasis, Institut National de la Santé et de la Recherche Médicale (INSERM) Unité Mixte de Recherche (UMR)1163, Paris, France; ^4^ Institut National de la Santé et de la Recherche Médicale (INSERM) U978, Université Paris 13 Sorbonne Paris Nord, Unité de Formation et de Recherche (UFR) Santé Médecine et Biologie Humaine (SMBH), Bobigny, France; ^5^ Institute of biological Sciences, Mohammed VI Polytechnic University (UM6P), Ben-Guerir, Morocco

**Keywords:** Fc receptors, inflammation, signaling, intracellular trafficking, insulin-regulated aminopeptidase

## Abstract

The function of intracellular trafficking in immune-complex triggered inflammation remains poorly understood. Here, we investigated the role of Insulin-Regulated Amino Peptidase (IRAP)-positive endosomal compartments in Fc receptor (FcR)-induced inflammation. Less severe FcγR-triggered arthritis, active systemic anaphylaxis and FcεRI-triggered passive systemic anaphylaxis were observed in IRAP-deficient *versus* wild-type mice. In mast cells FcεRI stimulation induced rapid plasma membrane recruitment of IRAP-positive endosomes. IRAP-deficient cells exhibited reduced secretory responses, calcium signaling and activating Syk^Y519/520^ phosphorylation albeit receptor tyrosine phosphorylation on β and γ subunits was not different. By contrast, in the absence of IRAP, SHP1-inactivating phosphorylation on Ser^591^ that controls Syk activity was decreased. *Ex-vivo* cell profiling after FcγR-triggered anaphylaxis confirmed decreased phosphorylation of both Syk^Y519/520^ and SHP-1^S591^ in IRAP-deficient neutrophils and monocytes. Thus, IRAP-positive endosomal compartments, in promoting inhibition of SHP-1 during FcR signaling, control the extent of phosphorylation events at the plasma membrane and contribute to setting the intensity of immune-complex triggered inflammatory diseases.

## Introduction

Insulin-regulated aminopeptidase (IRAP) is a widely expressed type II transmembrane protein with multiple functions. IRAP was initially described to colocalize with the glucose transporter Glut4 in endosomal compartments in adipocytes and to promote Glut4 translocation to the plasma membrane after stimulation with insulin (1). This detaches IRAP+ endosomes from their cytoskeletal anchor to which IRAP is bound *via* its N-terminal cytoplasmic tail (2, 3). In dendritic cells (DCs), the C-terminal aminopeptidase activity of IRAP trims peptides for MHC class I cross-presentation following recruitment of IRAP+ endosomes to phagosomes (4), a process which involves STIM1-dependent Ca^2+^ signaling (5). The aminopeptidase activity is also involved in the cleavage/inactivation of various peptide hormones including for example oxytocin or vasopressin (6, 7). In addition, IRAP+ recycling endosomes have emerged as compartments that regulate cell signaling in immune cells. IRAP controls TLR9 signaling by anchoring through its cytosolic tail TLR9+ endosomes to the actin cytoskeleton *via* binding to the formin FHOD4. This slows down TLR9 relocation into signal-competent lysosomes, while in the absence of IRAP TLR9 trafficking to lysosomes is increased thereby promoting signaling (8). The boost in inflammatory signaling may have detrimental effects as shown for example after infection with *Pseudomonas aeruginosa* (8). Contrasting with its role in TLR9 signaling, IRAP is capable to increase activation of T lymphocytes through the antigen T cell receptor (TCR). This involves formation of an intracellular endosomal signaling platform that amplifies TCR signaling (9). In line with this observation, TCR signaling requires vesicular compartments and the Golgi GMAP210 tethering protein recruiting LAT to TCR-activation sites at the immune synapse (10, 11). Likewise, the TCR zeta subunit, the Src-related tyrosine kinase Lck and LAT are recruited from distinct vesicular compartments to generate signal-competent nanoterritorries (12). The dependency of these events to IRAP remains to be explored.

IRAP may also play a role in signaling of receptors for the Fc domain of immunoglobulins (FcR). FcR are widely expressed in immune cells linking adaptive humoral immunity to cells of innate immunity such as mast cells, neutrophils, monocytes and macrophages (13–16). Like the TCR, and with the exception of the ITIM-bearing inhibitory receptor FcγRIIB, FcR belong to the family of immunoreceptors bearing a tyrosine-based activation motif (ITAM) contained either in the cytoplasmic tail of the ligand binding α subunit in single chain receptors or in associated β and γ signaling subunits. Activation of FcR by immunoglobulin (Ig) and antigen (Ag) complexes can promote phagocytosis, favor antigen presentation and induce the release of a large set of inflammatory products, cytokines and chemokines that contribute to the inflammatory and immune responses (17, 18). Their inappropriate activation can also play an important role in the development of inflammatory and autoimmune diseases (13, 19, 20).

Although FcR signaling at the plasma membrane has been worked out in quite detail the contribution of intracellular trafficking steps and of intracellular membrane pools to the signaling process remains largely unknown (13, 18, 19, 21). In FcεRI-bearing mast cells, IRAP was shown to localize to a VAMP2+ recycling compartment distinct from secretory granules (22). Upon crosslinking the FcεRI with IgE and Ag these IRAP+ compartments rapidly relocate to the plasma membrane. This contrasts with transferrin receptor (TfR)+ recycling endosomes known to cycle constitutively. IRAP relocation is insensitive to PI3K and PKC inhibition and requires calcium release from internal stores but not extracellular calcium influx (22). Hence, the authors speculated that relocation of IRAP+ endosomes may facilitate cell activation through recruitment of essential cell signaling components.

Therefore, we investigated the role of IRAP in experimental models of FcR-dependent acute and chronic inflammatory diseases such as IgE- and IgG-dependent anaphylaxis and IgG-dependent arthritis. Our findings show that IRAP amplifies FcR-induced responses as IRAP-deficient mice exhibit a less severe disease. We demonstrate that IRAP promotes the phosphorylation of several signaling effectors through inactivation of signal-regulating SHP-1 phosphatase thereby driving an enhanced calcium response and inflammatory mediator secretion.

## Materials & methods

### Cell isolation

Bone marrow-derived mast cells (BMMCs) were obtained by extracting bone marrow from femurs of 8 to 12-week-old C57B/6J and IRAP-deficient mice as described (23). Cells are cultured at 37°C, 5% CO_2_ in IMDM Glutamax medium HEPES 25 mM supplemented with 15% fetal bovine serum (FBS, GIBCO), 25 IU/mL of Penicillin and 25 μg/mL of Streptomycin, 1% nonessential amino acid (NEAA), 1 mM Sodium pyruvate, murine IL-3 (10 ng/mL) (Peprotech # 213-13-200UG), murine SCF (10 ng/mL) (Peprotech # 250-03-200UG) and β-mercaptoethanol 50µM. Cell differentiation into mast cells is complete after four weeks of culture. Medium is changed once a week by collection of non-adherent cells and resuspension in fresh medium. From the third week on, cells are seeded at 1,5x10^6^/mL and medium is changed twice a week. To generate peritoneal-derived mast cells (PDMCs), peritoneal lavage cells of C57B/6J or IRAP-deficient mice were grown in the same conditions as BMMCs.

### Analysis of cell purity

Purity of the mast cell cultures was confirmed by double positivity for anti-c-kit and anti-FcϵRI antibody labeling. Cells were washed twice in FACS buffer (PBS containing 1% BSA and 0.05% sodium azide) and incubated for 10 min at 4°C with 20 μL of home-made blocking Fc receptor solutions containing 100 μg/mL of polyclonal mouse IgGs, 100 μg/mL of polyclonal rat IgGs (Jackson Immunoresearch), 10 μg/mL of Armenian Hamster IgGs (Innovative Research, Inc) and 10 μg/mL of rat anti-mouse CD16/32 (clone 2.4G2, BioXcell). Cells were then incubated in dark for 30 min at 4°C with the indicated coupled antibodies or the related isotype controls: Alexa Fluor^®^ 647-conjugated anti-mouse FcϵRIα (Biolegend #134310), Alexa Fluor^®^ 647 Armenian Hamster IgG Isotype Ctrl (Biolegend #400924), APC-eFluor780 conjugated anti-mouse c-kit (Invitrogen #47-1171-82), Rat IgG2b kappa Isotype Ctrl APC-eFluor780 (Ebioscience #47-4031-82). After two washes in FACS buffer, cells were resuspended in 500 μl of FACS buffer and analyzed using a Fortessa flow cytometer (Becton Dickinson). Results were analyzed using the FlowJo software (Ashland, OR, USA). Cells were used for the various experiments after 4 to maximum 10 weeks of culture.

### Mice

IRAP-deficient mice on the Sv129 background were obtained from S. Keller (University of Virginia) and were backcrossed nine times to C57BL/6/J mice obtained from Janvier (St. Quentin-Fallavier, France) (2). C57BL/6 human FcγRIIA^Tg^ mice expressing the WT human FcγRIIA were purchased from Jackson Laboratory (JAX, Bar Harbor, ME, USA). IRAP-deficient FcγRIIA^Tg^ were obtained intercrossing FcγRIIA^Tg^ mice with IRAP-deficient mice. All mice were in the C57BL/6 background, of female gender and between 8-12 weeks old. Mice carrying the FcγRIIA transgene and littermates were used as heterozygous animals. Mice were bred and maintained at the mouse facilities of the Bichat Medical School campus. All animal experiments were performed in accordance with the French Council of Animal Care guidelines and national ethical guidelines. The study was approved by the local ethical committee (comité d’éthique en expérimentation animale, Faculté de Médecine Site Bichat Université Paris Diderot) and by the Department of Research of the French government under the animal study proposal numbers APAFIS# 14682 and 14156.

### IgE-induced passive systemic anaphylaxis

C57B/6J and IRAP-deficient mice (8 to 12 weeks old) were injected i.v. with mouse monoclonal anti-DNP IgE (clone H1 DNP-ϵ-26, 20 μg/mouse) purified from ascitic fluid (24, 25) and a thermal probe (model IPTT-300, PLEXX, Elst, The Netherlands) was placed under the dorsal skin of the mice. Twenty-four hours later they were challenged by i.v. administration of 500 μg DNP-HSA antigen (Sigma-Aldrich, St. Quentin Fallavier). For control experiments unsensitized mice were also directly challenged i.v. with 2 mg histamine (Sigma-Aldrich H7125-1G). The drop-in body temperature was measured using a wireless reader for thermal probes (model IPTT-300, PLEXX). When mice recovered the normal body temperature (≈ 80 minutes), 50 μL of blood were collected in heparin and plasma was used to quantify released mast cell protease 1 using a mouse MCPT-1 ELISA kit according to the manufacturer (Thermo Fisher Scientific #88-7503-22).

### IgG-induced active systemic anaphylaxis

FcγRIIA^Tg^ and FcγRIIA^Tg^ IRAP-deficient mice (8-12 weeks old) were immunized i.p. with normal rabbit IgG (0.5 mg/20 g body weight, Sigma Aldrich) in Complete Freund’s Adjuvant (Sigma-Aldrich #F5881). At day 6 a thermal probe (model IPTT-300, PLEXX, The Netherlands) was placed under the dorsal skin of the mice and one day later mice were challenged i.v. with rabbit IgG (100 μg/mouse). The drop in body temperature was measured using a wireless reader for thermal probes (PLEXX, Elst, Netherlands). When mice recovered the normal body temperature (≈ 60 minutes), 50 μL of blood were collected in heparin and blood cells were analyzed using a MS9-5S reader to obtain platelet counts (Melet Schloesing Laboratoires). Plasma was also used to quantify of mouse anti-rabbit IgG using ELISA plates precoated with rabbit IgG (Bethyl Laboratories #E90-131). After sample binding and washes bound mouse IgG was detected using a biotinylated anti-mouse IgG detection antibody according to the manufacturer’s instructions (Bethyl Laboratories #E90-131). In a separate series of experiments blood samples were also collected (5 minutes after challenge) for phosflow analysis.

### Mouse collagen antibody-induced arthritis model

Arthritis was induced using the Arthrogen-CIA^®^ Arthritogenic Monoclonal Antibody kit (Chondrex, Inc. cat.53100). Mice were injected i.v. with 5 mg of an anti-CII Ab cocktail (Day 0) followed by 50 μg LPS (i.p.) 4 days later. Arthritis scores were determined and graded blindly as described https://www.chondrex.com/documents/Scoring-System.pdf from day 5 to 8. Paw thickness was measured with a pocket thickness gauge (Mitutoyo, Paris France). On day 8, animals were sacrificed and hind paws and knees were fixed in formalin.

### Cell stimulation

BMMCs from C57B/6J and IRAP-deficient mice were incubated overnight with IgE-anti-DNP (ascitic fluid diluted 1 x 10^7^) in complete medium without IL-3 and SCF. For degranulation assays cells were washed twice in Tyrode’s buffer (NaCl 137 mM, KCl 5 mM, glucose 5.6 mM, CaCl2 1.8 mM, MgCl2 1 mM, BSA 0,5 mg/mL, HEPES 20 mM) and were then stimulated in Tyrode’s buffer with indicated doses of antigen DNP-HSA at 37°C for indicated times. The stimulation was stopped by cooling the cell suspension in ice. For cytokine release assays and analysis of phosphoproteins (phosflow and Western Blot) cells were stimulated in complete medium after addition of indicated doses of antigen DNP-HSA at 37°C for indicated times before collection of cell supernatants and cell lysis, respectively.

### Degranulation assays

IgE-sensitized mast cells were either sham-stimulated with PBS or were stimulated with indicated doses of antigen (DNP-HSA) in Tyrode buffer (20 mM HEPES pH7,2, 137 mM NaCl, 5mM KCl, 1mM MgCl_2_, 1,8 mM CaCl_2_, 5,6 mM Glucose, 0,5 mg/mL BSA). Mast cell degranulation was assessed by measurement of the release of the granule-stored enzyme β-hexosaminidase at 45 minutes of stimulation as described (26). Briefly, in a 96-well plate, 25μl of unstimulated or stimulated cell supernatants were added to 50 μl of β-hexosaminidase substrate solution containing para-nitrophenyl-N-acetyl-β-D-glucosaminide (Sigma-Aldrich) in 0.05 M Citrate buffer, pH 4,5. The total amount of contained enzyme contained in cells was evaluated using supernatant of unstimulated cells after lysis in 0,3% Triton-X100. After incubation for 1 hour and 30 minutes at 37°C the reaction was stopped by addition of 150 μl of 0.2M glycine, pH 10.7. The optical density (OD) was read with a TECAN SPARK 10M reader at 405 nm with wavelength correction set at 570 nm. The percentage of β-hexosaminidase released was then calculated relative to its total amount in non-stimulated cells. Degranulation was also evaluated by surface expression of CD63 using flow cytometry. IgE-sensitized mast cells were either not stimulated or were stimulated with indicated doses of antigen (DNP-HSA) for 10 min. Cells were fixed in 4% paraformaldehyde, incubated at 4°C with with Alexa Fluor^®^ 647-conjugated anti-mouse FcϵRIα (Biolegend #134310), Alexa Fluor^®^ 647 Armenian Hamster IgG Isotype Ctrl (Biolegend #400924), APC-eFluor780 conjugated anti-mouse c-kit (Invitrogen #47-1171-82), Rat IgG2b kappa Isotype Ctrl APC-eFluor780 (Ebioscience #47-4031-82), PE-conjugated anti-mouse CD63 (Biolegend #143903), PE Rat IgG2a, κ Isotype Ctrl (Biolegend #400507). Cells were resuspended in 500 μl of FACS buffer and analyzed using a Fortessa flow cytometer (Becton Dickinson). Results were analyzed using the FlowJo software (Ashland, OR, USA).

### Chemokine/cytokine ELISA assays

The supernatant of stimulated BMMCs was collected and after appropriate dilution the secretion of CCL2 and IL-6 was quantified by commercial ELISA kits according to the manufacturer’s instructions: mouse-CCL2 ELISA kit (PEPROTECH #900-K126), mouse IL-6 ELISA kit (R&D system #DY406-05).

### Calcium flux measurements

3x10^6^ IgE-sensitized BMMCs were loaded with 5 mg indo-1 (Invitrogen, Thermo Fisher) in RPMI medium supplemented with 10mM HEPES pH 7 for 30 min at 37°C. After washing, cells were allowed to adjust in medium supplemented with 10mM HEPES pH 7 and 5% FCS for 30 min at 37°C. Cells were washed and resuspended in RPMI medium supplemented with 5% FCS prior to the addition of 30 ng/mL DNP-HSA to induce calcium flux measured by using a FACSAria flow cytometer (BD Biosciences) to monitor the stimulation-dependent changes in fluorescent emission ratio (405 nm/475 nm). After 5 minutes of stimulation with DNP-HSA, Ionomycin at final concentration of 1 µM was added. Time-course analysis was performed using the FlowJo software package (Treestar, Ashland, Ore).

### Immunoblotting

Unstimulated and stimulated cells (1x10^6^ cells/mL), after stopping the reaction with ice-cold PBS, were washed twice in PBS. Cells were then lysed directly in 1x SDS Sample Buffer at 95°C in the presence of 5mM β-mercaptoethanol. Samples were resolved by SDS polyacrylamide gel electrophoresis, transferred to nitrocellulose membranes and immunoblotted with primary human pSyk^Y525/526^ rabbit mAb (CellSignaling #2710S) (also recognizing mouse pSyk^Y519/520^) followed by Protein A-HRP (Biorad #170-6522). Membranes were developed by SuperSignal kit West Femto Maximum Sensitivity Substrate (Thermo Scientific #34096). Non-phosphorylated controls were obtained after stripping of the membranes of the first-round antibodies with Restore Western Blot stripping buffer (Thermo Scientific #21059) followed by reblotting with Syk Rabbit Ab (CellSignaling #2712S) and Protein A-HRP as before.

### Phosflow analysis

After stimulation BMMCs or blood mouse cells were fixed by addition of BD lysis/fix buffer (BD #349202). The surface staining was done with indicated conjugated antibodies to gate BMMCs, blood neutrophils and blood monocytes respectively. The following Abs were used: PE anti-mouse FcεRIα (Biolegend #134307), Brilliant Violet 421™ anti-mouse CD45 (Biolegend #103134), Brilliant Violet 421™ Mouse IgG1, κ Isotype Ctrl (Biolegend #400158) Brilliant Violet 605™ anti-mouse/human CD11b (Biolegend #101257), Brilliant Violet 605™ Rat IgG2b, κ Isotype Ctrl (Biolegend #400657), APC/Fire™ 750 anti-mouse Ly-6G/Ly-6C (Gr-1) (Biolegend #108456), APC/Fire™ 750 Rat IgG2b, κ Isotype Ctrl (Biolegend #400670), PE anti-mouse CD115 (CSF-1R) (Biolegend #135506), PE Rat IgG2a, κ Isotype Ctrl (Biolegend #400508), PerCP/Cyanine5.5 anti-mouse CD19 (Biolegend #115534), PerCP/Cy5.5 Rat IgG2a, κ Isotype Ctrl (Biolegend #400532), Alexa Fluor^®^ 700 Hamster anti-Mouse CD3e (BD #557984), Alexa Fluor^®^ 700 Hamster IgG2, λ Isotype Ctrl (BD #557985). Cells were then permeabilized with Cytofix/CytopermTM (BD #554714) and intracellular staining was performed using indicated conjugated antibodies and appropriate corresponding Ig-isotype controls. Abs used: human Syk (Tyr525 +Tyr526) Antibody (Bioss #bs-3434R-A488) (recognizing Tyr519 + Tyr520 in mouse sequence), Rabbit mAb IgG isotype control Alexa 488 (Cellsignaling 2975S), Syk (SantaCruz sc-1240 AF488), normal mouse IgG2a AF488 (SantaCruz sc-3891), LAT Tyr200 (Bioss bs-10128R-A488) (recognizing Tyr185 in mouse sequence), Rabbit IgG isotype control AF488 (Bioss bs-0295P-A488), LAT (SantaCruz sc-373706 AF790), Normal Mouse IgG2b (SantaCruz sc-516630 AF790), p-p38 Tyr182 (SantaCruz sc-166182 AF647), Normal mouse IgG2a AF647 (santacruz sc-24637), p38 (Santacruz sc-7972 AF405), Normal mouse IgG1 AF405 (Santacruz sc-45069), SHP-1 Signalway Antibody C32280-AF647), Rabbit mAb IgG XP^®^ Isotype Ctrl Alexa Fluor^®^ 647 Conjugate (Cell Signaling #2985S), Alexa Fluor^®^ 647 Anti-SHP1 (phospho Ser591) (Bioss # Bs-5578R), Rabbit mAb IgG XP^®^ Isotype Ctrl Alexa Fluor^®^ 647 Conjugate (Cell Signaling #2985S). After washing, cells were analyzed by using a Fortessa flow cytometer (Becton Dickinson) and FlowJo software (Ashland, OR, USA). Beads used for the single stainings: OneComp eBeads™ Compensation Beads (Invitrogen #01-1111-42).

### Histological analysis

To obtain mast cell counts, sections of tissues embedded in paraffin were incubated with toluidine blue (Sigma-Aldrich #T3260) for 10 minutes, rinsed, dried and mounted with mounting medium (Eukitt #EUK100). For histological analysis of arthritis, formalin-fixed hind paws and knees from the mice were decalcified and embedded in paraffin. Sagittal sections were stained with hematoxylin and eosin. Images were taken with a 40X lens using a slide scanner (Aperio ScanScope).

### Electron microscopy analysis

BMMCs were fixed in 4% formaldehyde/PBS at RT for 1 hour, and stored at 4°C. After washing, cells were post-fixed with 1% glutaraldehyde in PBS for 1h at RT and then rinsed and pelleted in gelatin 12%. Gelatine pieces containing cells were post-fixed with 1% osmium tetroxide reduced with 1.5% potassium ferrocyanide in PBS for 1h, progressively dehydrated in ethanol, and embedded in low-viscosity epoxy resin (Agar Scientific Ltd). Blocks were cured at 60°C for 24h. 70-nm-thin sections were cut using an EM UC6 ultramicrotome (Leica), mounted on copper grids, and stained with uranyl acetate and lead citrate. Sections were examined with a 120 kV TEM (Tecnai 12, Thermo Fischer Scientific) equipped with a 4K CDD camera (Oneview, Gatan).

### Immunofluorescence microscopy

Small squares (about 0.8cm x 0.8cm) were drawn in super frost glass slides by a hydrophobic pen and 50μL of fibronectin (10μg/mL) (Sigma #F1141) was added for 30 minutes at 37°C to the slides. IgE-sensitized cells were resuspended at 1.5 x 10^6^ cells/mL in complete medium (without IL-3 and SCF) containing 1 mM MnCl_2_ to favour adherence. 75μL of the cell suspension was added to the fibronectin coated slides and cells were allowed to adhere for 45 minutes at 37°C. After washing, cells were stimulated by addition of 50µL of medium containing 30 ng/mL of DNP-HSA at 37°C for the appropriate time. 4% paraformaldehyde was added directly to the slide for 15min at 37°C. Cells were permeabilized in PBS-Tween 0.1% for 10 minutes at room temperature before addition for 2 hours of primary unconjugated antibodies diluted at the concentration indicated by the manufacturer in PBS-BSA 0.2%-Tween 0.1%. Abs used: IRAP (D7C5) XP^®^ Rabbit mAb (Cell signaling #6918S), LifeSpan Bioscience. WA, USA. Cat#LS- C149375, Syk (Tyr525 + Tyr526) Antibody (Bioss #bs-3434R), Rabbit IgG LifeSpan Bioscience. WA, USA. Cat#LS- C149375, Lyn polyclonal antibody (Invitrogen PA5-27361), Syk Rabbit Ab (CellSignaling #2712S), LifeSpan Bioscience. WA, USA. Cat#LS- C149375, IRAP (3E1) Mouse mAb (Cell Signaling #9876S), mouse IgG affinity purified (Innovative Research Inc, IMSIGGAP10MG-819). After washes, cells were incubated one hour in the dark with appropriate secondary antibodies at 10 µg/mL in PBS-BSA 1% - Tween 0.1%. Abs used: Goat anti-Mouse IgG (H+L) Cross-Adsorbed Secondary Antibody, Alexa Fluor 488 (Life technologies #A11001), Goat anti-Rabbit IgG (H+L) Cross-Adsorbed Secondary Antibody, Alexa Fluor 594 (Invitrogen #A11012). After washing the cells were fixed 15 minutes in Paraformaldehyde 4%/PBS followed by incubation in 50mM NH_4_Cl/PBS to quench background. Finally, cells were mounted in Prolong gold antifade reagent in the presence of DAPI (Invitrogen, Thermo Fisher Scientific). The acquisition of the images was performed with the 63X lens with a confocal Leica sp8 microscope and data processing was done using Image J software.

### Gating strategy of delimitation of the region of interest used for quantitative analysis of immunofluorescence experiments

Marker colocalizations were evaluated using only non-saturated images and the ImageJ software. A manual threshold was established for each channel before image analysis. Individual cells were delimitated with the freehand selection tool, and considered as ROI in ImageJ. For colocalization studies, all images were first translated to a binary image (black pixel intensity = 0; white pixel intensity = 1). The binary images for independent channels were multiplied to create a mask that encompasses the pixels present in both channels. The areas of pixels for each color and of mask pixels were calculated with ImageJ. The percentage of pixels for one color that colocalized with pixels for another color was calculated as the ratio: sum of area of pixels in the mask divided by sum of area of pixels from the first color. For the quantification of PM and intracellular fluorescent signals, two regions of interest were delimited with freehand selection tool and the area of fluorescent signal in these regions were measured using Image J.

### Immunoprecipitation

1 x 10^7^ IgE-sensitized BMMCs/condition were stimulated with 200 ng/mL of DNP-HSA. After indicated time points, the stimulation was stopped by adding ice-cold PBS and placed in ice. Cells were washed twice in ice-cold PBS and lysed for 30 min in 1mL HEPES 25mM, NaCl 140mM, Triton 0.2%, EDTA (1mM) and phosphatase/protease inhibitor cocktail (NaF 5 mM, Na_3_VO_4_1 mM, PepstatinA, Aprotinin, AEBSF, Leupeptin). FcεRI was then immunoprecipitated by incubation with protein G beads (Cytiva #17061801) and 3µg of anti-FcεRI β chain mAb (clone 30.9) (27) in rotation at 4°C. After washing 5 times with HEPES 25mM, NaCl 140mM, Triton 0.05%, EDTA 1mM, samples were resuspended in 1X sample buffer and subjected to immunoblotting with mouse anti-phosphotyrosine-HRP (clone Py20) (Southern Biotech 1400-05). The amount of immunoprecipitated receptor was determined after stripping of the membranes of the first-round antibodies with Restore Western Blot stripping buffer (Thermo Scientific #21059) and reblotting with anti-FcεRI β chain mAb and Protein A-HRP as before.

### Statistical analysis

All numbers of repeats for experiments and statistical tests carried out are provided in the respective figure legends. Statistical analysis was performed using the Graphpad Prism v8.00 software (GraphPad Software, Inc., La Jolla, CA, USA).

## Results

### IRAP is involved in FcR-mediated anaphylaxis

**Figure 1:**
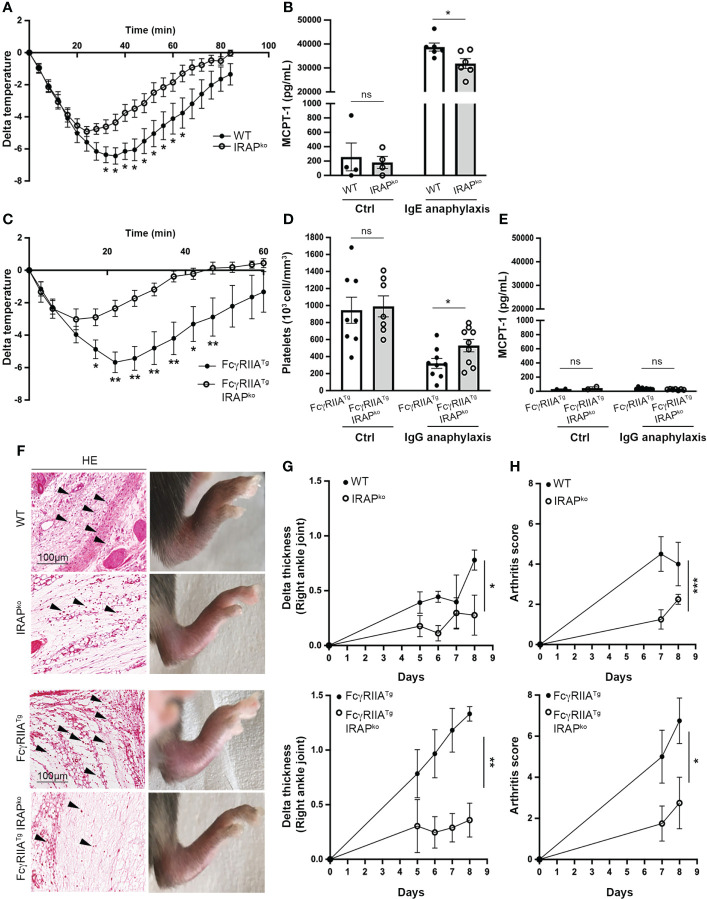
IRAP-deficient mice show less severe IgE- and IgG-induced anaphylactic reactions and experimental arthritis. **(A)** 24h after sensitization with anti-DNP IgE wild-type **(**WT) and IRAP-deficient (IRAP^KO^) mice were challenged with antigen (DNP-HSA) to induce passive systemic anaphylaxis (PSA). The drop in body temperature was evaluated. Data presented are the mean ± s.e.m. with 9 mice (pooled from 3 experiments). **(B)** Released serum MCPT-1 chymase collected at the end of temperature measurements was evaluated in the two groups of mice as well as in unsensitized control mice. **(C)** WT FcγRIIA^Tg^ and FcγRIIA^Tg^ IRAP^KO^ mice were immunized with rabbit IgG and 7 days later mice were challenged with rabbit IgG to induce active systemic anaphylaxis (ASA). The drop in body temperature was evaluated. Data are presented as mean ± s.e.m. with 6 mice (pooled from 2 experiments). **(D)** ASA was monitored by evaluating plasma platelet counts collected at the end of temperature measurements in the two groups of mice as well as in non-immunized control mice. Data are the mean ± s.e.m. **(E)** Serum MCPT-1 chymase collected at the end of temperature measurements was measured in the two groups of mice as well as in non-immunized control mice. Data are the mean ± s.e.m. **(F)** WT and IRAP^KO^ or WT FcγRIIA^Tg^ and FcγRIIA^Tg^ IRAP^KO^ mice were injected with an anti-collagen type II Ab cocktail (day 0) followed by injection of LPS (day 4). Photographs shows representative hematoxylin/eosin (HE) staining of ankle sections as well as the macroscopic appearance of hind legs for each genotype at day 8. **(G)** Arthritis development was monitored by measuring paw thickness starting 5 days after injection of the Ab cocktail. **(H)** Arthritis scores were also evaluated according to the provided scoring system (Chondrex). Data are the mean ± s.e.m. from 4 to 7 mice/group. Statistical analysis was done using the two-way ANOVA followed by Sidak’s *post-hoc* test **(A, C, G, H)** or the unpaired Student’s t test **(B, D, E)**. *: P < 0.05; **: P < 0.01; *** P < 0.001; ns, not significant.

To explore whether IRAP modulates FcR-induced inflammatory responses we analyzed mast cell- and IgE receptor (FcεRI)-dependent passive systemic anaphylaxis (PSA) comparing WT and IRAP-deficient mice. Before starting the experiments, we first verified that IRAP-deficient mice did not show any abnormalities in mast cell frequency. Histologic analysis and quantification of mast cells in tissues revealed that skin, ear and tongue from both types of mice had similar numbers of mast cells indicating that IRAP did not impact mast cell development and tissue distribution (**Supplementary Figure 1A**). Likewise, ultrastructural analysis by electron microscopy of cultured mast cells (BMMC) did not reveal differences in cell morphology and granular appearance (**Supplementary Figure 1B**) and the total content of β-hexoaminidase, an enyme stored in secretory granules, was comparable **(** **Supplementary Figure 1C** **)**. We also found that both genotypes responded equally to a challenge with histamine, the major mediator of mast cell driven anaphylaxis, ruling out a decreased responsiveness of IRAP-deficient mice to mast cell histamine (**Supplementary Figure 1D**). PSA experiments were then conducted by sensitizing mice i.v. with IgE anti-DNP followed by a challenge 24h later with specific antigen (DNP-HSA). The ensuing drop in body temperature was then monitored continuously over 80 min after challenge. IRAP-deficient mice showed a less severe anaphylaxis as they exhibited a significantly reduced drop in body temperature when compared to WT mice, favoring a more rapid recovery (**Figure 1A**). The PSA reaction was associated with an increase in serum levels of mast cell protease 1 (MCPT1) confirming the implication of mast cells, but this increase was lower in IRAP-deficient mice suggesting a reduced activation of mast cells (**Figure 1B**).

To investigate whether IRAP affects signaling of other ITAM-bearing FcR we analyzed active systemic anaphylaxis (ASA) induced by IgG. IgG-mediated anaphylaxis, involving the IgG receptors (FcγR) FcγRIIA and FcγRIII in FcR-humanized mouse models, can be the cause of certain drug-induced anaphylaxis and of serious side effects in monoclonal IgG antibody therapies (28–30). Based on their reported strong response profile, we used WT or IRAP-deficient mice transgenic for human FcγRIIA (FcγRIIA^Tg^) and a previously described model of active systemic anaphylaxis (ASA) where, in addition to monocytes and neutrophils, activation of platelets plays an important role (31). FcγRIIA^Tg^ WT and IRAP-deficient mice were immunized with normal rabbit IgG and seven days later mice were challenged with rabbit IgG to induce ASA. Like before, the anaphylactic response was measured by the drop in body temperature monitored over 60 min. As expected (31), the FcγRIIA^Tg^ in the WT background developed rapid and severe hypothermia (**Figure 1C**) as well as a marked thrombocytopenia (**Figure 1D**). Like for the IgE-induced PSA, FcγRIIA^Tg^ in the IRAP-deficient background were significantly less impacted from IgG-mediated ASA as they exhibited a less severe drop in temperature and in platelet counts (**Figures 1C, D**). In agreement with reports that IgG-mediated ASA does not involve mast cells (32), MCPT1 levels increased neither in FcγRIIA^Tg^ WT nor in FcγRIIA^Tg^ IRAP-deficient mice (**Figure 1E**). As IRAP was shown to be involved in Ag cross-presentation (4), we performed control experiments analyzing the immune response mounted in FcγRIIA^Tg^ WT and FcγRIIA^Tg^ IRAP-deficient mice in this model. No significant difference was observed in the levels of anti-rabbit IgG produced by the two genotypes (**Supplementary Figure 1E**).

To extend the role of IRAP to a more chronic inflammatory setting, we used the collagen antibody-induced arthritis (CAIA) mouse model, where arthritis is passively induced using anti-collagen type II mAbs and LPS. IgG-containing immune complexes that can bind to FcγR and proinflammatory cytokines are crucial players in the pathogenesis of CAIA-induced arthritis (33, 34). Since the disease is enhanced in mice transgenic for human FcγRIIA (35), we induced CAIA both in WT and humanized FcγRIIA transgenic mice deficient or sufficient for IRAP by treating them with a cocktail of anti-collagen mAb (day 0) and LPS (day 4). Arthritis development was monitored from the day they started to develop the disease, at day 5 up to day 8. (**Figure 1F**) shows hematoxylin and eosin stains of ankle sections from these mice, as well as representative photographs of the swelling of hind paws observed for the analyzed genotypes at the day of sacrifice. Pictures reveal the typical features of arthritis development with ankles exhibiting significant inflammatory cell infiltration in the synovium and development of paw swelling. Paw swelling and arthritis scores were significantly reduced in IRAP-deficient mice when compared to their WT or FcγRIIA^Tg^ counterparts (**Figures 1G, H**). Together, these results revealed an important role of IRAP in setting the intensity of FcR-mediated inflammation.

### IRAP is involved in FcεRI-mediated MC degranulation and cytokine production

**Figure 2:**
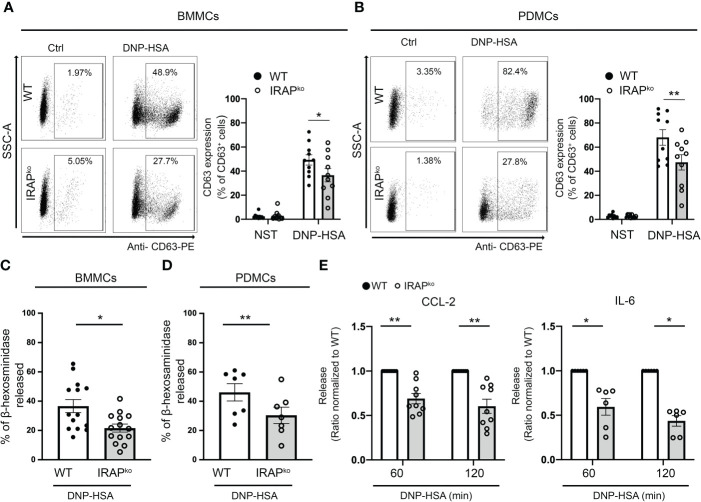
IRAP-deficient mice exhibit a diminished degranulation response and cytokine/chemokine production in BMMCs and PDMCs. WT and IRAP-deficient (IRAP^KO^) BMMCs **(A, C)** and PDMCs **(B, D)** were sensitized with anti-DNP IgE. **(A, B)** Degranulation was monitored by flow cytometry determining CD63 expression (% expression is indicated) as a surrogate marker of mast cell degranulation 10 min after addition of PBS (basal expression) or antigen (30 ng/mL of DNP-HSA). A representative experiment (left panel) and the quantitative analysis of indicated number of experiments are shown (right panel). **(C, D)** Degranulation was also determined by measuring the net release of the granular enzyme β-hexosaminidase in BMMCs and PDMCs after stimulation with IgE/Ag for 45 minutes in indicated number of experiments. **(E)** IgE-sensitized BMMCs were also evaluated for their ability to secrete CCL2 and IL6 after stimulation with IgE/Ag for 60 and 120 min as indicated. Data shown in **(A–D)** are the mean ± s.e.m of indicated experiments. Statistical analysis was done using an unpaired Student’s t test. Data in **(E)** have been normalized by setting WT values to 1. The range of maximal release was between 2,3 to 83,2 ng/mL for CCL-2 and 61,7 to 3781 pg/mL for IL-6. Statistical analysis was done using a Wilcoxon Test. No differences in basal secretion in the absence of Ag was noted between the genotypes: CCL2 WT 1,95 ± 0,98 ng/mL; IRAP KO 2,46 ± 0,63 ng/mL; p = 0,75 and IL6 WT 9,5 ± 6,6 pg/mL; IRAP KO 5,6 ± 4,0 pg/mL; p = 0,63; unpaired Student’s t test; *: P < 0.05; **: P < 0.01.

Based on these *in vivo* results we next explored whether IRAP is able to enhance FcR-mediated signaling by focusing on FcϵRI-mediated mast cell degranulation known to represent the prominent effector mechanism in IgE-dependent PSA. IgE-dependent activation of WT and IRAP-deficient bone marrow-derived mast cells (BMMCs) and peritoneal-derived mast cells (PDMCs) was first analyzed by measuring CD63 expression, a surrogate marker of mast cell degranulation (36), on gated BMMCs and PDMCs expressing equal levels of c-Kit and FcεRI (**Supplementary Figure 2A**). Results show that cells readily increase CD63 cell surface expression after FcεRI-induced stimulation. While no differences in basal expression were observed, we found that, after stimulation, the CD63 expression was significantly lower in IRAP-deficient mast cells when compared to WT cells (**Figures 2A, B**). These results were confirmed by analyzing release of the granule-stored enzyme β-hexosaminidase. In both types of primary mast cells, although the total content of stored β hexosaminidase in non-stimulated cells was similar (**Supplementary Figure 1C**), after stimulation with IgE/Ag, the release was significantly decreased in IRAP-deficient cells when compared to WT cells (**Figures 2C, D**). Of note, neither the expression of c-KIT nor of FcεRI was affected by the absence of IRAP in BMMCs and PDMCs (**Supplementary Figures 2A, B**, left panel). As FcεRI stimulation also induces cytokine/chemokine secretion, we analyzed the release of IL-6 and CCL-2 in FcεRI-activated BMMCs from WT and IRAP-deficient mice. Like the degranulation response, secretion of CCL-2 and IL-6 was significantly impaired in the absence of IRAP (**Figure 2E**). These results confirm the *in vivo* data supporting that the presence of IRAP can enhance the secretion of inflammatory products in FcεRI-stimulated mast cells.

### IRAP regulates the phosphorylation of signaling effectors upon FcεRI activation

**Figure 3:**
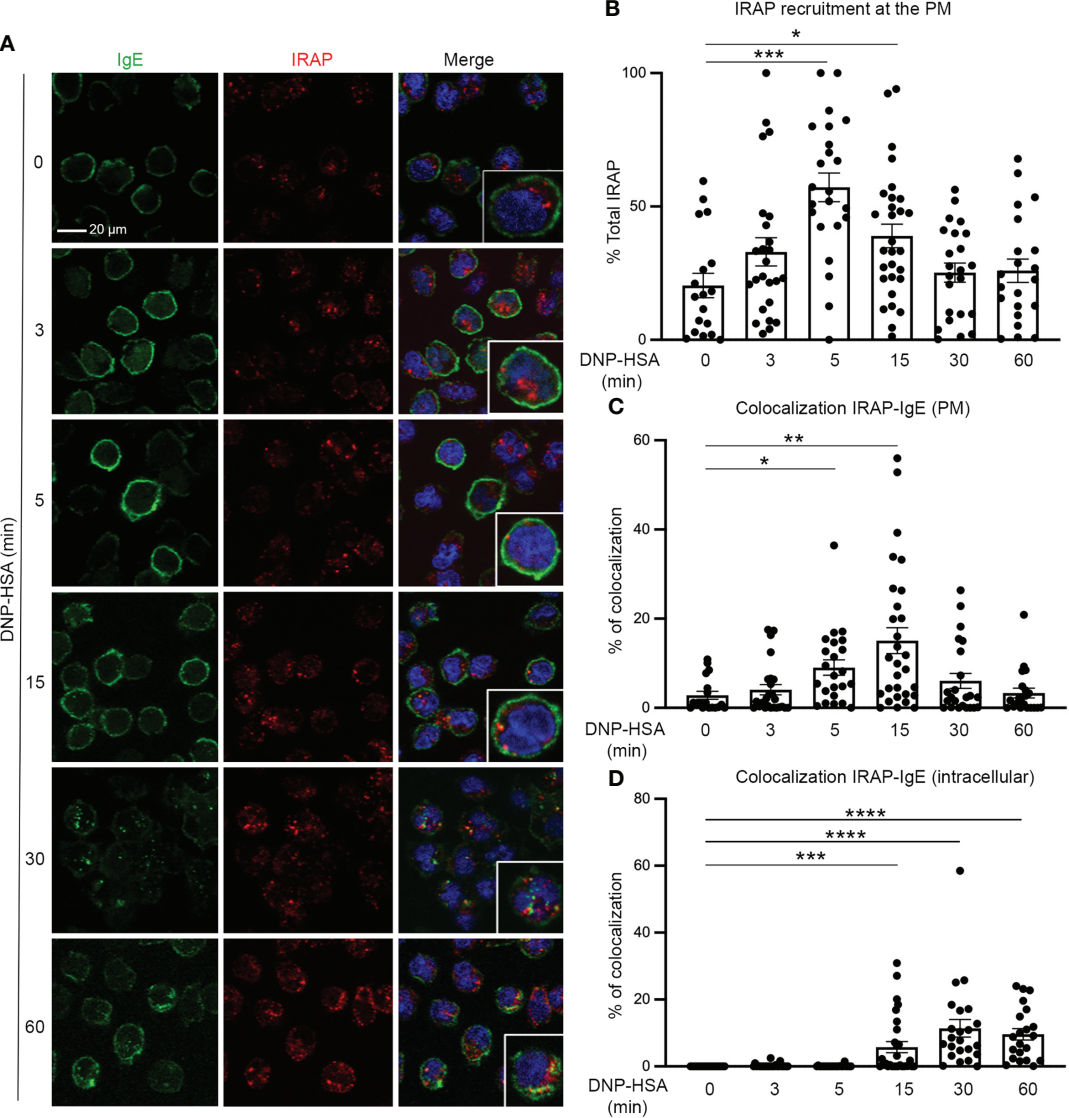
IRAP rapidly relocates to the plasma membrane and partly colocalizes with IgE after stimulation of mast cells with Ag. **(A)** WT BMMCs were sensitized with anti-DNP IgE and were then plated on fibronectin-coated glass coverslips. Cells were stimulated for indicated time points with antigen DNP-HSA. After fixation and permeabilization cells were stained with DAPI (blue), anti-IgE (green) and anti-IRAP (red) and analyzed by confocal microscopy. Images show representative sections with multiple cells for IgE and IRAP single staining as well as the merge of all colors. In the merge an insert with an enlarged cell is shown to evidence colocalization. **(B)** Quantitative analysis of plasma membrane recruitment was determined as described in Materials & Methods. **(C)** Quantitative analysis of colocalization of IRAP and IgE at the plasma membrane (PM) or **(D)** intracellularly following internalization of FcεRI-bound IgE. Note that internalization of FcεRI-bound IgE starts at 15 min. **(B, C, D)** Statistical analysis for plasma membrane recruitment and colocalization of IgE with IRAP was done using the one-way ANOVA followed by a Kruskal Willis post-test. Data shown are presented as the mean ± s.e.m of indicated individual cells (pooled from 3 experiments). *: P < 0.05; **: P < 0.01; ***: P < 0.001; ****: P < 0.0001.

To explore the mechanisms by which the amino-peptidase regulates FcεRI-mediated secretory events, we verified previous data (22) indicating that IRAP+ recycling endosomes are recruited to the plasma membrane in mast cell upon FcεRI stimulation. The results shown in **Figures 3A, B** confirm that in resting BMMCs IRAP mainly localizes to intracellular vesicular structures previously defined as IRAP+ recycling endosomes, with only a minor part (~ 20%) being present at the plasma membrane (22). Upon IgE-dependent stimulation, a sizable fraction (~ 60%) of IRAP+ endosomes rapidly relocated to the plasma membrane reaching a maximum after 5 min of stimulation before starting to get internalized again. Colocalization analysis showed that, after stimulation, IRAP partly colocalized with FcεRI at the plasma membrane increasing at early time points (up to 15 min) and then intracellularly at later time points as the FcεRI was endocytosed in IRAP+ compartments (**Figures 3C, D**).

**Figure 4:**
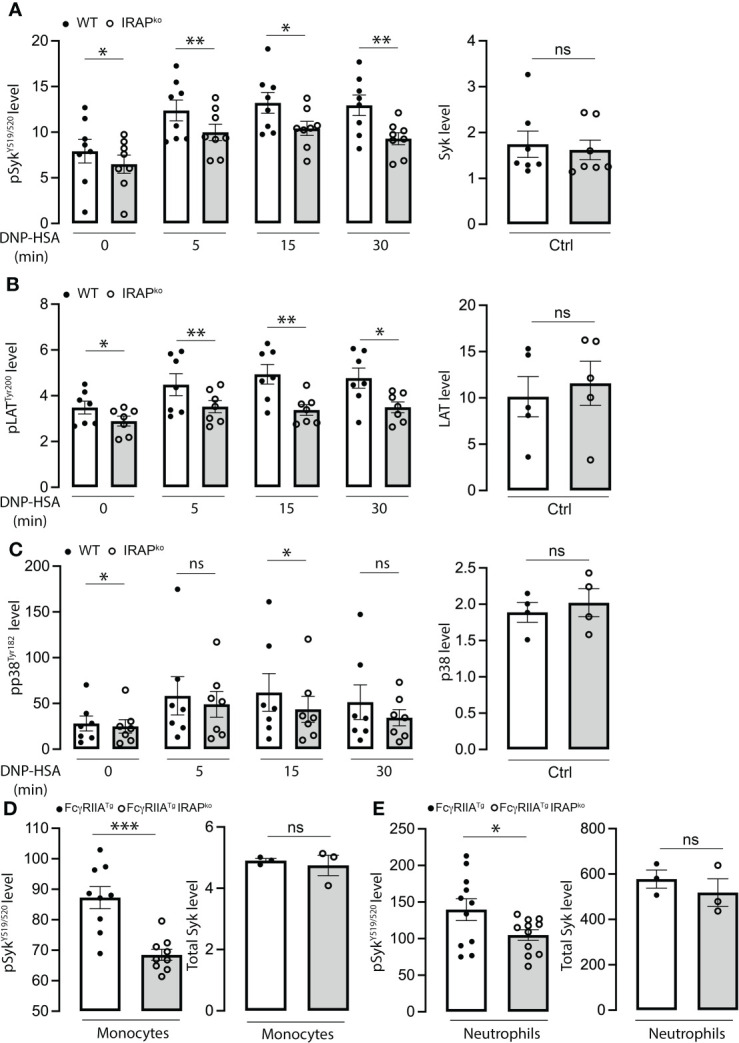
Diminished phosphorylation response of signaling effectors in FcεRI-stimulated BMMCs and IgG stimulated neutrophils and monocytes in the context of IRAP-deficiency. **(A–C)** WT and IRAP-deficient (IRAP^KO^) BMMCs were sensitized with anti-DNP IgE for 24 hours before stimulating them with specific antigen (DNP-HSA). After indicated time points, stimulation was arrested by adding fixation and permeabilization buffer. Phosflow analysis of signaling effectors (left panel) was assessed using anti-pSyk^Y519/520^ **(A)**, anti-pLAT^Y200^ **(B)** and anti-pp38^Y182^ **(C)**. Total levels of proteins in non-stimulated cells were also analyzed using anti-Syk, anti-LAT and anti p38 (right panels). Phosphorylation levels were determined as the ratio of the gMFI of samples divided by the gMFI of the FMO of the respective sample and represent the mean ± s.e.m of indicated experiments. Statistical analysis was done using a Student’s test. **(D, E)** Phosflow analysis of Syk phosphorylation at Y^519/520^ was also determined on *ex vivo* IgG-stimulated neutrophils and monocytes during ASA. Cells were accessed directly from the blood 5 min after initiation of ASA. The gating strategies for analysis of BMMCs, neutrophils and monocytes and representative examples of Ab staining are shown, respectively, in (**Supplementary Figures 2B, C**). Data shown are analyzed as in **(A–C)** and presented as the mean ± s.e.m of indicated experiments. Statistical analysis was done using a Student’s test. *: P < 0.05; **: P < 0.01; *** P < 0.001. ns, not significant.

IRAP recruitment at the plasma membrane was reported to be independent of FcεRI-stimulated extracellular calcium influxes as well as of PI3K and PKC activation, supporting the hypothesis that it may foster signaling events upon plasma membrane recruitment (22). Based on this, we analyzed a variety of tyrosine phosphorylated proteins using Phosflow experiments. Our quantitative analysis (**Figures 4A-C**) (for gating strategy of BMMCs and representative examples of Ab staining see (**Supplementary Figure 2B**) shows that several proteins (pSyk^Y519/5520^, pLAT^Y200^ and pp38^Y182^) exhibited reduced phosphorylation in the context of IRAP-deficiency in kinetic experiments, while basal expression of these proteins did not differ between the two genotypes in BMMCs. To confirm these results in cells obtained *ex vivo* and to extend them to FcγR, we analyzed neutrophils and monocytes as effector cells in IgG-induced ASA in FcγRIIA^Tg^ mice which, contrary to mast cells, can be directly accessed from the circulation. After initiation of ASA, blood was drawn at 5 min and gated permeabilized neutrophils and monocytes were analyzed using anti-pSyk^Y519/520^ (for gating strategy and representative examples of Ab staining see (**Supplementary Figure 2C**). Results show that pSyk is significantly decreased both in neutrophils and monocytes of IRAP-deficient FcγRIIA^Tg^ *versus* IRAP-sufficient FcγRIIA^Tg^ mice, while basal expression of these proteins did not differ between the two genotypes (**Figures 4D, E**, right panels). These experiments identify IRAP as a modulator of cell signaling through FcϵRI and FcγRIIA, *in vitro* and *in vivo*.

**Figure 5:**
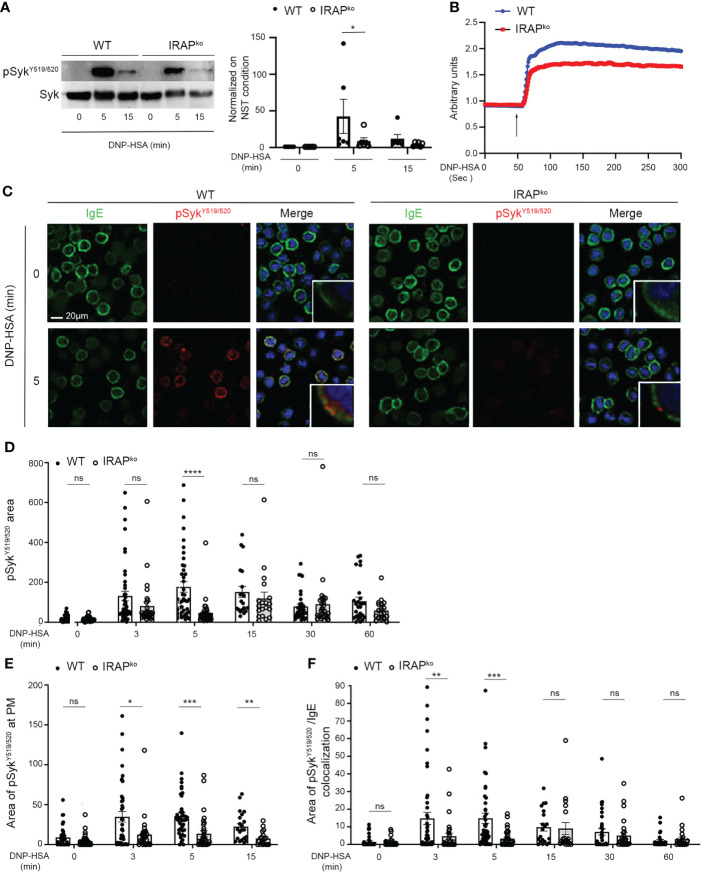
IRAP-deficient BMMCs show diminished phosphorylation of Syk at the plasma membrane where it colocalizes with IgE. **(A)** WT and IRAP-deficient (IRAP^KO^) BMMCs were sensitized with anti-DNP IgE and challenged with antigen (30 ng/mL of DNP-HSA). Stimulation was arrested at indicated time points and cellular lysates were prepared in SDS sample buffer. Western Blot analysis (left panel) show pSyk staining (anti-pSyk^Y519/520^) and total Syk determined after stripping. Quantitative data (right panel) are the ratio between pSyk/Syk and were normalized by setting non stimulated values to 1. They represent the mean ± s.e.m of indicated experiments. Statistical analysis was done using the Wilcoxon test. **(B)** Anti-DNP IgE sensitized WT (blue) and IRAP^KO^ BMMCs (red) were loaded with indo-1 and stimulated after addition (arrow) of 30 ng/mL of DNP-HSA. The fluorescence emission ratio for Ca^2+-^bound/Ca^2+-^free indo-1 was measured. Similar results were obtained in three independent experiments. **(C)** Anti-DNP IgE sensitized WT and IRAP^KO^ BMMCs were plated on fibronectin-coated glass coverslips. Cells were stimulated with 30 ng/mL of DNP-HSA for indicated time points. After fixation and permeabilization cells were stained with DAPI (blue), anti-IgE (green) and anti-pSyk^Y519/520^ (red) as indicated. Cells were analyzed by confocal microscopy. Images show representative sections with multiple cells for IgE and pSyk^Y519/520^ single staining as well as the merge of all colors. In the merge an insert with an enlarged cell membrane is shown to evidence colocalization. For a complete kinetic analysis see **(** **Supplementary Figure 4** **)**. **(D)** The total amount of pSyk^Y519/520^ in both genotypes was determined for all time points examined. **(E)** The amount of pSyk^Y519/520^ appearing at the plasma membrane within the first 15 min after stimulation was quantified. **(F)** The areas of colocalization between pSyk^Y519/520^ and IgE during the entire time of stimulation were quantified. **(D–F)** Data shown are presented as the mean ± s.e.m of indicated individual cells analyzed (pooled from 3 different experiments). Statistical analysis was done using the two-way ANOVA followed by Sidak’s *post-hoc* test. *: P < 0.05; **: P < 0.01; *** P < 0.001; **** P < 0.0001. ns, not significant.

Given that Syk is a central effector in FcϵRI induced signaling, we confirmed that Syk phosphorylation on Y519/520 was minored in IRAP-deficient BMMCs when analyzed by Western blotting at 5 min following stimulation with specific antigen (**Figure 5A**). As Syk activation is crucial for calcium mobilization, we next analyzed calcium fluxes in BMMCs from WT and IRAP-deficient mice. (**Figure 5B**) shows a representative tracing of the calcium response in FcεRI-stimulated mast cells from both genotypes. They are concordant with the Syk phosphorylation data as the response is less prominent in IRAP-deficient cells. To analyze whether IRAP promoted phosphorylation of Syk locally at the plasma membrane, we examined its phosphorylation status using confocal microscopy. Whilst analysis of total Syk exhibited a diffuse staining pattern throughout the cytoplasm and in the nucleus as reported before (37, 38), we did not observe any noticeable differences in the staining pattern between resting and FcεRI-stimulated cells from both genotypes (**Supplementary Figure 3**). In contrast, FcεRI stimulation induced a rapid transient increase of pSyk^Y519/520^ (peaking at 5 min) (**Figures 5C, D** and **Supplementary Figure 4** for a representative complete kinetic analysis) that was largely present at the plasma membrane (**Figure 5E**) and that largely correlated with the kinetics of IRAP recruitment at the plasma membrane (**Figure 3B**). In agreement with data that Syk gets recruited to the FcεRI, a sizable amount of pSyk^Y519/520^ colocalized in areas that stained for FcεRI also peaking at 5 min of stimulation (**Figure 5F**). The Syk phosphorylation appearing at the plasma membrane was much less prominent and its association with FcεRI appeared delayed in IRAP-deficient cells (**Figures 5E, F**). Syk phosphorylation diminished at later time points, although levels remained higher than in non-stimulated cells. However, differences between the two genotypes vanished at these later timepoints, where total phospho-Syk was largely observed intracellularly coinciding with the internalization of aggregated FcϵRI (**Figures 5C–F**; **Supplementary Figure 4**).

**Figure 6:**
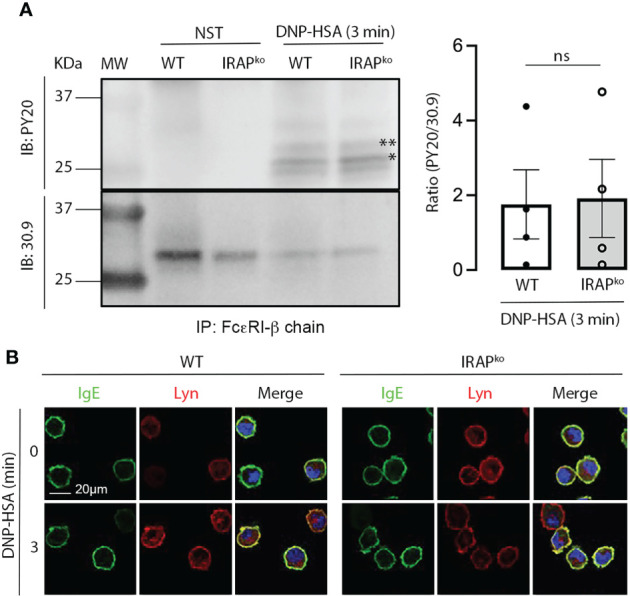
Absence of IRAP does not affect early signaling events mediated by Lyn kinase. **(A)** IgE-sensitized WT and IRAP-deficient (IRAP^KO^) BMMCs were either not stimulated or stimulated with antigen (30 ng/mL of DNP-HSA) before being lysed and immunoprecipitated with anti-FcεRIβ chain mAb. Levels of co-immunoprecipitated phospho-β chain (**) and phospho-γ chain (*) in non-reduced gels were determined using anti-phospho-tyrosine (PY20) Ab. Total immunoprecipitated FcεRI receptors were determined after stripping and reblotting with anti-FcεRβ chain mAb. A representative blot and quantitative analysis of indicated number of experiments are shown in the left and right panels, respectively. Statistical analysis was done using a Student’s t-test. **(B)** WT and IRAP-deficient (IRAP^KO^) BMMCs were sensitized with anti-DNP IgE and were then plated on fibronectin-coated glass coverslips. Cells were stimulated with 30 ng/mL of DNP-HSA for indicated time points. After fixation and permeabilization cells were stained with DAPI (blue), anti-IgE (green) and anti-Lyn (red) Abs as indicated. Cells were analyzed by confocal microscopy. Images show representative sections (out of 2 experiments) with multiple cells for IgE and Lyn single staining as well as the merge of all colors. ns, not significant.

**Figure 7:**
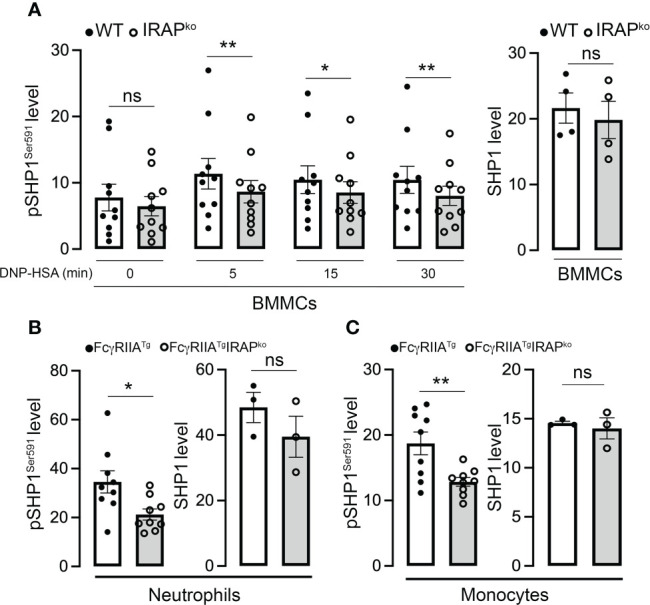
IRAP-deficient cells show less SHP1-inactivating phosphorylation on Ser591. **(A)** Anti-DNP IgE sensitized WT and IRAP-deficient (IRAP^KO^) BMMCs were stimulated with specific antigen (30 ng/mL of DNP-HSA) for indicated time points and levels of the SHP1^S591^ phosphorylation response were determined at indicated time points by phosflow analysis using anti-SHP1^S591^ (left panel). Total levels of proteins of resting cells were analyzed using anti-SHP1 (right panel). **(B, C)** SHP1 phosphorylation on Ser591 was also determined on IgG-stimulated neutrophils **(B)** and monocytes **(C)** during ASA analyzed ex vivo using phosflow analysis. Cells were accessed directly from the blood 5 min after initiation of ASA. The gating strategies for analysis of BMMCs, neutrophils and monocytes and representative examples of Ab staining are shown, respectively in (**Supplementary Figures 2B, C**). Phosphorylation levels in **(A–C)** were determined as the ratio of the gMFI of samples divided by the gMFI of the Isotype of the respective sample and represent the mean ± s.e.m of indicated experiments. Statistical analysis was done using a Student’s test. *: P < 0.05; **: P < 0.01; ns, not significant.

Enhanced Syk phosphorylation at the plasma membrane in WT cells could be the result of i) an enhanced phosphorylation of the γ subunit of FcεRI to which Syk is recruited and activated (39, 40) ii) of an increased presence of local signaling effectors, but also iii) of a decrease in phosphatase activity that negatively regulates Syk tyrosine phosphorylation (41, 42). We therefore tested these hypotheses. Whereas receptor β and γ subunits became rapidly phosphorylated after stimulation, no differences were noted between WT and IRAP-deficient BMMCs (**Figure 6A**). This is in agreement with our data showing that Lyn, the kinase responsible for β and γ chain phosphorylation (43) largely localized at the plasma membrane with FcεRI-bound IgE before and after short-term stimulation, with no noticeable differences in colocalization between the two genotypes (**Figure 6B**). We also looked whether some of the involved early signaling effectors exhibited a colocalization with IRAP+ endosomes in resting and stimulated cells. However, we did not detect a significant colocalization neither with Syk nor with Lyn nor with Fyn neither at early nor at later time-points of stimulation (**Supplementary Figures 5A–C**). Finally, we examined SHP1 phosphatase activity by measuring its phosphorylation on S591, which is known to be associated with its inactivation, thereby promoting ITAM signaling (44, 45). As shown in our Phosflow analysis (**Figure 7A** and **Supplementary Figure 2B** for gating strategy and representative examples of Ab staining) FcεRI-stimulation of WT BMMCs promoted phosphorylation of SHP1 on S591, which was significantly decreased in IRAP-deficient BMMCs, while basal expression of SHP1 did not differ. Likewise, *ex vivo* analysis of neutrophils and monocytes shortly after induction of IgG-dependent ASA revealed an enhanced S591 phosphorylation when the cells were isolated from FcγRIIA^Tg^ mice in the WT-background as compared to FcγRIIA^Tg^ in the IRAP-deficient background, while basal expression of SHP1 did not differ (**Figures 7B, C** and **Supplementary Figure 2C** for gating strategy and representative examples of Ab staining). This supports that IRAP favors FcR ITAM signaling by restricting the activity of SHP1 phosphatase.

## Discussion

It has become clear that subcellular compartments such as endosomes and their intracellular trafficking represent important components of cell signaling. They participate in surface expression of receptors, organize specific signaling platforms in intracellular compartments or control the availability and/or localization of relevant signaling effectors (9, 46, 47). IRAP, an aminopeptidase localized to slow recycling endosomal compartments, has emerged as an important player in this concept. Besides its *bona fide* aminopeptidase activity, IRAP-mediated trafficking steps were demonstrated to control cell signaling through TLRs and the TCR in immune cells (8, 9). Like the TCR, FcR belong to the family of immunoreceptors implicated in diverse effector functions that contribute to the inflammatory and immune response (18). But their inappropriate activation by pathologic Ig immune complexes can also lead to autoimmune and chronic inflammatory diseases (18, 48, 49).

Based on previous indications that IRAP+ endosomal vesicles rapidly relocate to the plasma membrane upon FcεRI stimulation in mast cells (22), we explored here whether IRAP could be relevant in FcεR-mediated inflammation and signaling. Using an *in vivo* model of IgE-mediated PSA applied to WT and IRAP-deficient mice we demonstrate that in the absence of IRAP anaphylaxis was impaired, supporting that IRAP enhances FcεRI-induced inflammation and signaling. In addition, we extended the role of IRAP also to FcγR-driven inflammation as IgG-mediated ASA was also impaired in IRAP-deficient mice when compared to WT mice. The measured difference in the temperature drop was even higher when compared to IgE-mediated PSA. This was likely due to the fact that we used an active systemic anaphylaxis model that may involve several FcγR (17, 18). The role of IRAP in FcγR-driven inflammation was also confirmed in chronic models of FcγRIII- and transgenic human FcγRIIA-dependent collagen antibody-induced arthritis, as in the context of IRAP-deficiency mice similarly exhibited less severe phenotypes when compared to their WT counterparts. Together these data supported that IRAP acts as an amplifier of both FcεRI- and FcγR-mediated inflammatory responses *in vivo*.

To gain mechanistic insights, we analyzed FcR-mediated cell signaling focusing to a large part on FcεRI-stimulated responses in mast cells. We corroborated earlier reports (22) showing that FcεRI stimulation promotes the rapid recruitment of IRAP to the plasma membrane in BMMCs. A sizeable part (about 60%) of IRAP+ endosomal compartments rapidly relocated to the plasma membrane following FcεRI stimulation reaching a maximum at 5 min before getting internalized again at later time points. Following plasma membrane recruitment, IRAP partly (up to 20%) colocalized with FcεRI-bound IgE. This colocalization was also observed intracellularly once receptors get endocytosed starting at 15 min and persisting up to one hour in IRAP+ compartments. Although IRAP was partly colocalized with the receptor, we did not observe an interaction with the receptor in co-immunoprecipitation experiments. Previously, studies in mast cells had shown that the relocation of IRAP to the plasma membrane was insensitive to inhibitors of FcεRI signaling such as PKC and PI3K. It was also independent of extracellular calcium influx (22), while increases of intracellular calcium levels were required both for its plasma membrane recruitment in mast cells (22) and for supporting antigen phagosomal delivery during cross-presentation in dendritic cells (5). Thus, in contrast to the reported late endosomal/lysosomal function of IRAP in TLR and TCR signaling (8) (9), these data supported that IRAP may function at early signaling steps at the plasma membrane prior to PKC, PI3K activation or calcium fluxes from the extracellular milieu. Testing this more closely, we found that phosphorylation of several FcεRI proximal signaling effectors at the plasma membrane were impaired in the absence of IRAP. These signaling effectors included the tyrosine kinase Syk and a major Syk substrate, the adaptor LAT (50), as well as the downstream Syk-dependent signaling effector p38 MAPKinase (51, 52). In agreement with the central role of Syk in FcεRI signaling (50), IRAP-deficient mast cells exhibited a decreased calcium response as well as an impaired degranulation and secretion of CCL2 and IL6. The decrease in Syk phosphorylation was also confirmed *ex vivo* in IRAP-deficient monocytes and neutrophils after activation by IgG-induced ASA (29).

Previous data indicated that IRAP participates in cell signaling by its ability to direct intracellular trafficking. To regulate intracellular trafficking, the cytosolic domain of IRAP interacts with several cytoskeleton remodeling proteins (9) and with the retromer (53), a large protein complex that recycles vesicular cargos, avoiding their transport to lysosomes. By interacting with actin remodeling factors, in dendritic cells, IRAP was shown to maintain TLR9 together with its ligand in early endosomes impeding its trafficking to signal-competent lysosomes (54). In consequence, TLR9 signaling is increased in IRAP-deficient cells, in which TLR9 and its ligand are massively targeted to signal-competent lysosomes (8). In the case of T cells, IRAP interacts with the CD3ζ chain (CD247) of the TCR, which is the limiting component for TCR expression at cell surface (55). In basal conditions, CD247 is internalized by the same mechanisms as IRAP and is targeted to an intracellular endocytic pool, whose stabilization depends on IRAP (9). In the absence of IRAP, the intracellular CD247 pool is diminished and the cell surface amount of CD247 is increased, leading to increased levels of the entire TCR complex at plasma membrane. Despite increased expression of TCR complex at plasma membrane, IRAP-deficient T cells showed defective TCR signaling, leading to the hypothesis that the intracellular vesicles containing both IRAP and CD247 enable the assembly of an intracellular endosomal signaling platform that enhances TCR signaling (9).

Concerning FcεRI, we did not observe a differential expression and the receptor was endocytosed both in the presence and absence of IRAP. However, confocal analysis showed that the crucial signaling kinase Syk in its activated form (pSyk^Y519/520^) appeared more rapidly at the plasma membrane in WT cells when compared to IRAP-deficient cells, coinciding with the kinetics of plasma membrane recruitment of IRAP. This suggested that IRAP supported an early step in the signaling process at the plasma membrane. The enhancement of Syk phosphorylation was not due to a lesser expression of Syk at the plasma membrane, as we detected no differences of global Syk expression and Syk localization between WT and IRAP-deficient cells. We therefore analyzed possible pathways involved in the activation of Syk in mast cells. One crucial step represents its recruitment to the phosphorylated ITAM motif within the disulfide-linked FcεRγ chains which, like the phosphorylation of FcϵRIβ chain, relies on the Src-related kinase Lyn (39, 43). However, Lyn expression and localization showed no differences between the two genotypes. Likewise, the phosphorylation of FcϵRIβ and γ chains in activated BMMC, as a readout of Lyn activity, revealed no differences in immunoprecipitated receptors ruling out an effect of IRAP on the activity of this Src kinase. Although recruitment to the FcεRγ ITAMs represents an important step in the activation of Syk, its phosphorylation is not uniquely dependent on ITAM recruitment, a finding which is also consistent with the fact that Syk gets activated by ITAM-independent receptors (56). Other events such as direct phosphorylation by Lyn (40) and the activity of phosphatases, notably SHP1, have been reported to play important roles (56–58). As Lyn-dependent receptor subunit phosphorylation was not altered in the absence of IRAP, we focused our attention on SHP1. It has been reported that SHP1, during ITAM-dependent signaling, becomes inactivated through its phosphorylation on S591 thereby shifting the balance to a kinase-driven cell signaling response (45, 59). In agreement, our analysis confirmed that following aggregation of FcεRI, SHP1 rapidly becomes phosphorylated on S591. Yet, this phosphorylation was minored in IRAP-deficient mast cells. Similar data were obtained when we analyzed SHP1 phosphorylation on residue S591 in neutrophils and monocytes isolated shortly after the initiation of IgG-induced ASA. Here also S591 phosphorylation was less prominent when cells had been isolated from IRAP-deficient mice. Together these data indicated that the presence of IRAP increases FcεRI and FcγRIIA signaling by inactivating SHP1 phosphatase therby promoting the shift to a kinase-driven signaling cascade.

In conclusion, the data presented here demonstrate that FcR-induced signaling responses are enhanced in the presence of the multifunctional protein IRAP that localizes to early recycling endosomes in immune cells (9). In mast cells, FcεR engagement stimulated an enhanced and rapid redistribution of these IRAP+ endosomes to the plasma membrane. While this early recruitment did not impact very early observable events such as receptor phosphorylation mediated by Lyn kinase, it largely coincided with an enhanced presence of activated Syk at the plasma membrane and downstream events such as calcium fluxes, degranulation and cytokine secretion. Further analyses showed that this involved the ability of IRAP to favor inactivation of SHP1 phosphatase, thereby shifting the balance to a kinase driven response. *In vivo*, this was associated with enhanced inflammatory responses in IRAP-sufficient *versus* deficient mice both in FcεRI and FcγR-induced inflammatory disease models. Therefore, our results reveal that IRAP-positive recycling compartments actively participate in FcR signaling and contribute to setting the intensity of phosphorylation events at the plasma membrane and in consequence FcR-mediated inflammatory responses and diseases.

## Data availability statement

The raw data supporting the conclusions of this article will be made available by the authors, without undue reservation.

## Ethics statement

The study was approved by the local ethical committee: Comité d’éthique en expérimentation animale, Faculté de Médecine Site Bichat Université Paris Diderot) and by the Department of Research of the French government under the animal study proposal numbers APAFIS# 14682 and 14156.

## Author contributions

UB, LS and SM conceptualized the study, designed the experiments, interpreted the data, and wrote the manuscript. MBr, SV, DK, and CL conducted experiments. MBr, SV, and CL analyzed the data. GM and MBe helped with the analysis of the data and did manuscript corrections. All authors provided input on final manuscript. All authors contributed to the article and approved the submitted version.

## Funding

This work was funded by Inserm, CNRS, and Université de Paris. This work was also supported by the Investissements d’Avenir program ANR-19-CE15-0016 IDEA, ANR-11-IDEX-0005-02 (Sorbonne Paris Cite, Laboratoire d’excellence INFLAMEX) and ANR JC (ANR-17-CE17-0002-01). MBr received a doctoral fellowship from Inflamex (ANR-11-IDEX-0005-02 (Sorbonne Paris Cite, Laboratoire d’excellence INFLAMEX).

## Acknowledgments

We sincerely thank Dr. Nicolas Charles for his valuable suggestions throughout this work as well as Lucie Auguet for her valuable help in some of the experiments. We thank S. Keller (University of Virginia) for providing the IRAP-deficient mice. We acknowledge the ImagoSeine core facility of the Institut Jacques Monod, member of the France BioImaging infrastructure (ANR-10-INBS-04) and GIS-IBiSA for performing electron microscopy analysis.

## Conflict of interest

The authors declare that the research was conducted in the absence of any commercial or financial relationships that could be construed as a potential conflict of interest.

## Publisher’s note

All claims expressed in this article are solely those of the authors and do not necessarily represent those of their affiliated organizations, or those of the publisher, the editors and the reviewers. Any product that may be evaluated in this article, or claim that may be made by its manufacturer, is not guaranteed or endorsed by the publisher.
