# Supplementary material for: Insulin-regulated aminopeptidase contributes to setting the intensity of FcR-mediated inflammation
Source: Front Immunol. 2022 Oct 27;13:1029759. doi: 10.3389/fimmu.2022.1029759 (PMC9647545; doi:10.3389/fimmu.2022.1029759)
Supplement: Supplementary file 1 [file DataSheet_1.docx]

**Supplementary Material**

**Insulin-regulated aminopeptidase contributes to setting the intensity of FcR-mediated inflammation**

by

Manuela Bratti^1,2^, Shamila Vibhushan^1,2^, Cyril Longé^3^, Despoina Koumantou^1,2^, Gael Ménasché^3^, Marc Benhamou^1,2^, Nadine Varin-Blank^4^, Ulrich Blank^1,2^*^#^, Loredana Saveanu^1,2^* and Sanae Ben Mkaddem^4,5^ *

^1^Université Paris Cité, Centre de Recherche sur l'Inflammation, INSERM UMR1149, CNRS EMR-8252, Faculté de Médecine site Bichat, Paris, France

^2^Université Paris Cité, Laboratoire d'Excellence Inflamex, Paris, France.

^3^Université Paris Cité, Imagine Institute, Laboratory of Molecular basis of altered immune homeostasis, INSERM UMR1163, F-75015 Paris France.

^4^INSERM U978, Université Paris 13 Sorbonne Paris Nord, UFR SMBH, Bobigny, France

France

^5^ Institute of biological Sciences, Mohammed VI Polytechnic University (UM6P), 43150, Ben-Guerir, Morrocco

* co-senior authors; ^#^ corresponding author

Figure S1. Shows in control experiments that WT and IRAP deficient mice respond equally to a challenge of histamine, contain equal numbers of mast cells in tissues and mount similar humoral immune response.

Figure. S2. Shows gating strategies to measure CD63 expression and gating strategy for phosflow analysis of BMMCs and mouse blood neutrophils and monocytes after ASA.

Figure S3. Shows that WT and IRAP-deficient BMMCs have a similar staining pattern of Syk in resting and stimulated BMMCs.

Figure S4. Shows a representative complete kinetic confocal analysis of pSyk staining in WT and IRAP-deficient BMMCs.

Figure S5. Shows that Syk, Lyn and Fyn do not colocalize with IRAP in resting and stimulated BMMCs.


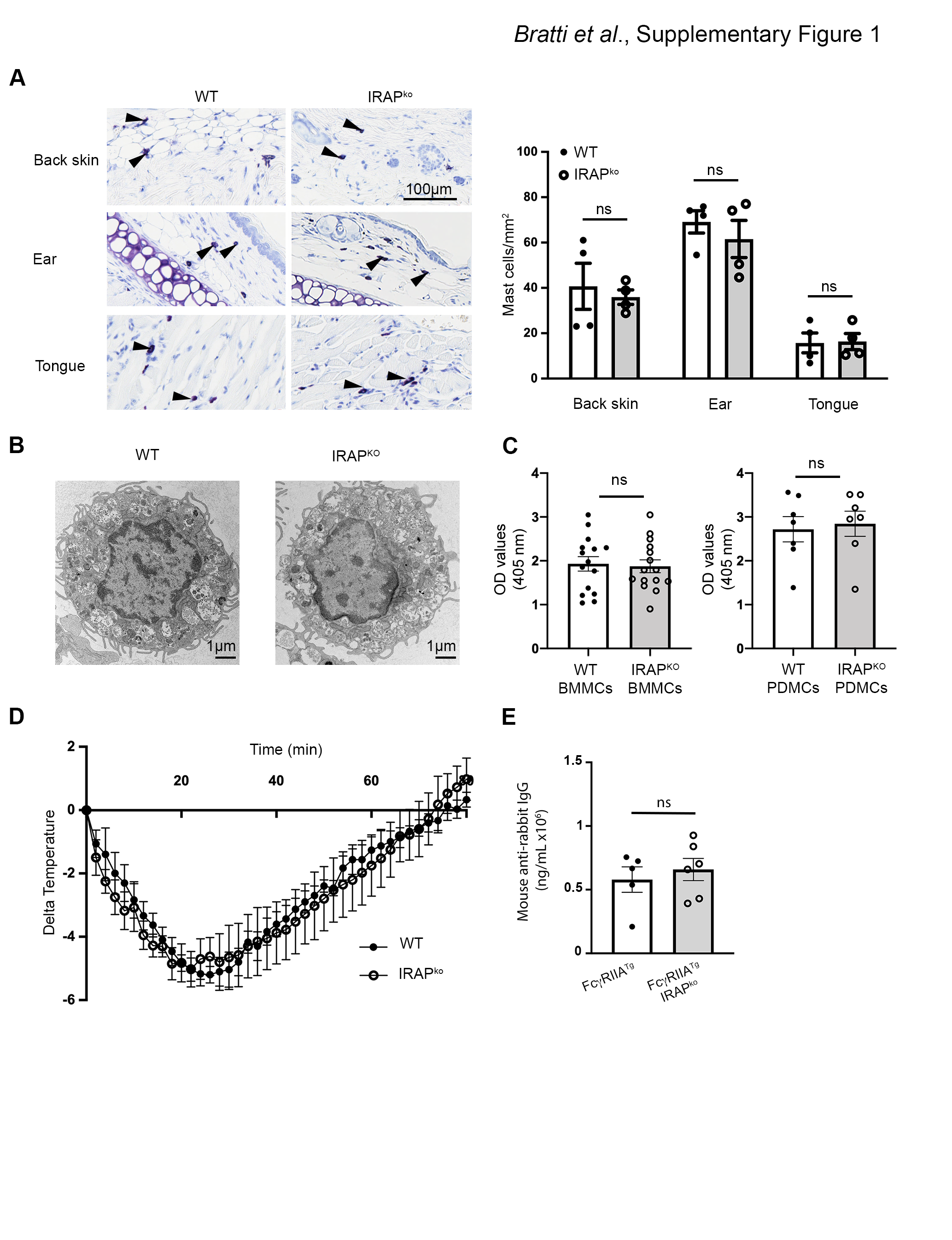
**Supplementary Figure 1:** Comparative analysis of WT and IRAP-deficient mice and mast cells. (A) Left panel shows toluidine blue stained mast cells in back skin, tongue and ear tissues from WT and IRAP-deficient (IRAP^KO^) mice. Right panel shows corresponding mast cell counts. Data represent the mean ± s.e.m from 4 mice. Statistical analysis: Student’s t test. ns = not significant. **(B)** Ultrastructural analysis by electron microscopy of cultured mast cells (BMMCs) from WT and IRAP^KO^ mice. (C) Total content granule-stored β-hexosaminidase from cultured mast cells (BMMC and PDMCs). Statistical analysis: Student’s t test. **(D)** WT and IRAP-deficient (IRAP^KO^) mice were challenged with histamine (2 mg) to induce passive systemic anaphylaxis (PSA). PSA was monitored by evaluating the drop in body temperature every 2 min using a wireless reader detecting a thermal probe placed under the dorsal skin of the mice 24 h prior to challenge. Data presented are the mean ± s.e.m. of 4 mice/group. Statistical analysis: two-way ANOVA followed by Sidak's post-hoc test. **(E)** FcγRIIA transgenic mice in the WT (FcγRIIA^Tg^) and IRAP-deficient background (FcγRIIA^Tg^ IRAP^KO^) were immunized i.p. with normal rabbit IgG (500 µg) emulsified in Complete Freund’s adjuvant. At day 7 blood was drawn and analyzed for produced anti-rabbit IgG by Elisa. Data are presented as mean ± s.e.m. with indicated numbers of mice. Statistical analysis: unpaired Student t test.


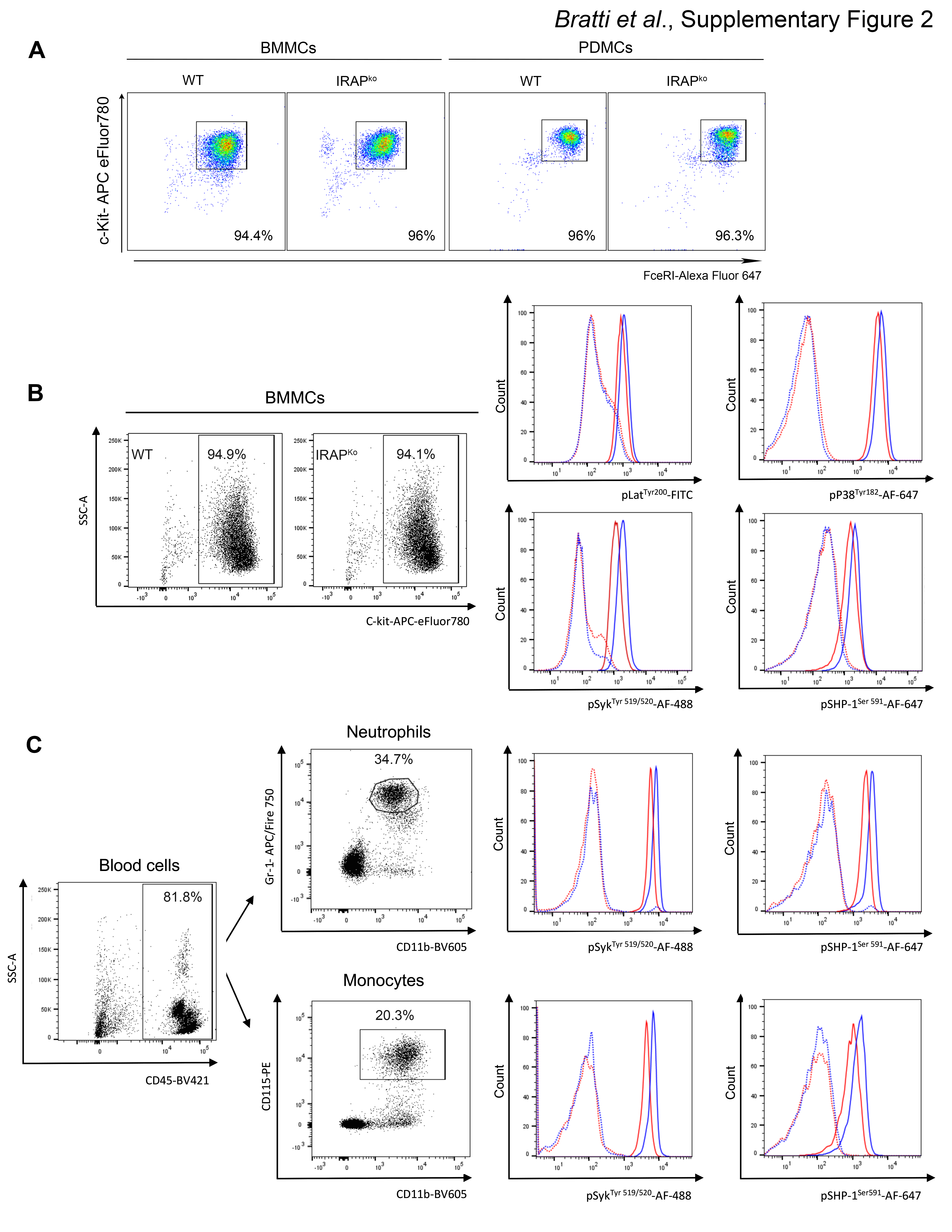


**Supplementary Figure 2:**  Gating strategy for determining CD63 expression and gating strategy used for phosflow analysis. **(A)** Representative flow cytometry gating strategy used for analysis of CD63 expression of WT and IRAP-deficient (IRAP^KO^) BMMCs and PDMCs. **(B)** Representative flow cytometry gating strategy used for phosflow analysis of WT and IRAP-deficient (IRAP^KO^) BMMCs (left panels) as well as a representative experiment of pLAT, pp38, pSyk and pSHP1 expression (right panels) of IgE sensitized WT and IRAP-deficient (IRAP^KO^) cells that have been stimulated with 30 ng/mL of Ag (DNP-HSA). **(C)** Representative flow cytometry gating strategy used for phosflow analysis of WT (FcγRIIA^Tg^) and IRAP-deficient (FcγRIIA^Tg^ IRAP^KO^) neutrophils and monocytes (left panels) as well as the histograms of a representative experiment of pSyk and pSHP1 expression (right panels) obtained from the blood of WT (FcγRIIA^Tg^) and IRAP-deficient (FcγRIIA^Tg^ IRAP^KO^) mice 5 min after initiation of ASA by challenge with rabbit IgG. Blue lines show the stimulated condition and red lines show the non-stimulated condition. Dotted lines represent the corresponding isotype control.


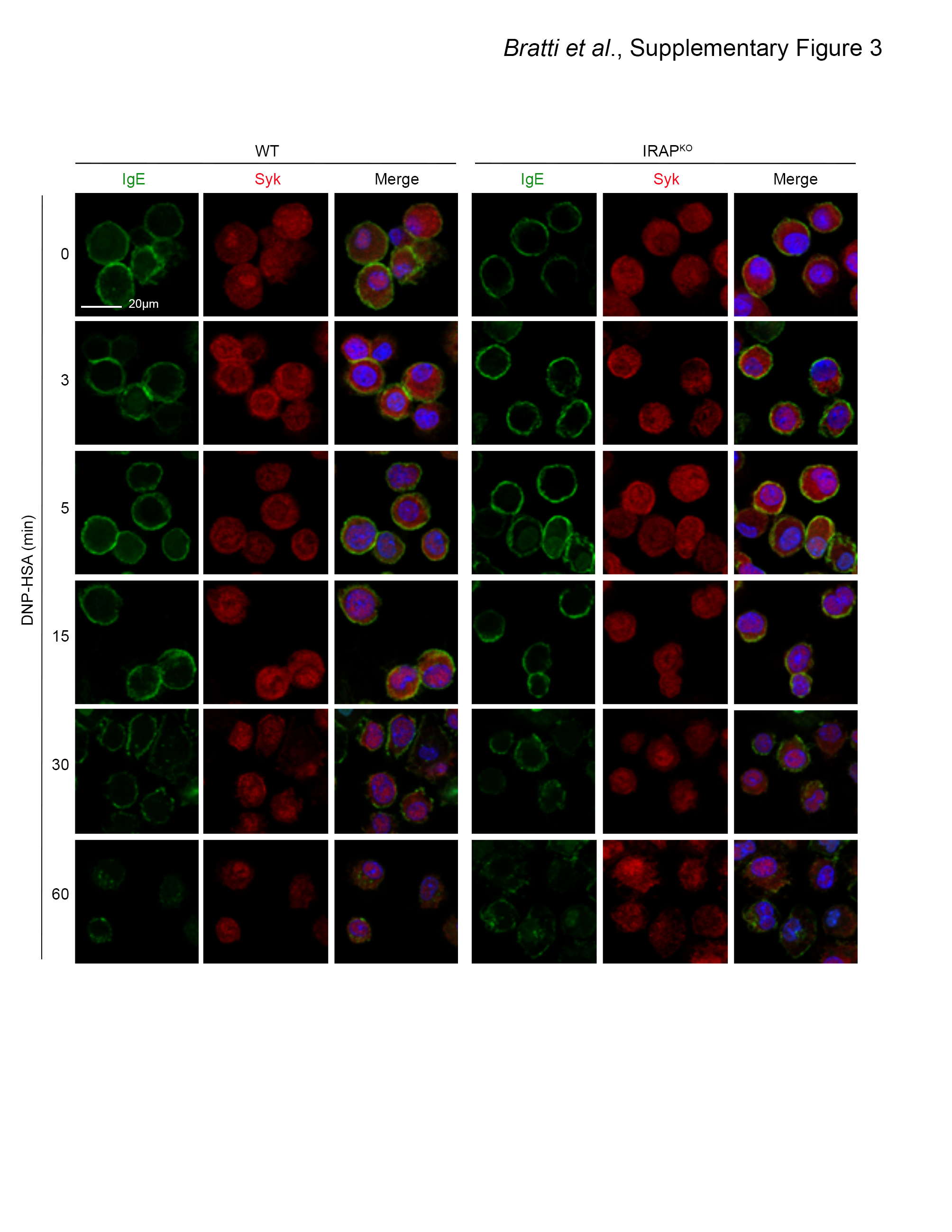


**Supplementary Figure 3:**  Similar staining pattern of Syk in resting and stimulated BMMCs. WT and IRAP-deficient (IRAP^KO^) BMMCs were sensitized with anti-DNP IgE for 24 hours and were then plated on fibronectin-coated glass coverslips. Cells were stimulated with DNP-HSA (30 ng/mL) for indicated time points. After fixation and permeabilization cells were stained with DAPI (blue), anti-IgE (green) and anti-Syk (red) Abs as indicated. Cells were analyzed by confocal microscopy. Images show representative sections (out of 2 experiments) with multiple cells for IgE and Syk single staining as well as the merge of all colors.

**
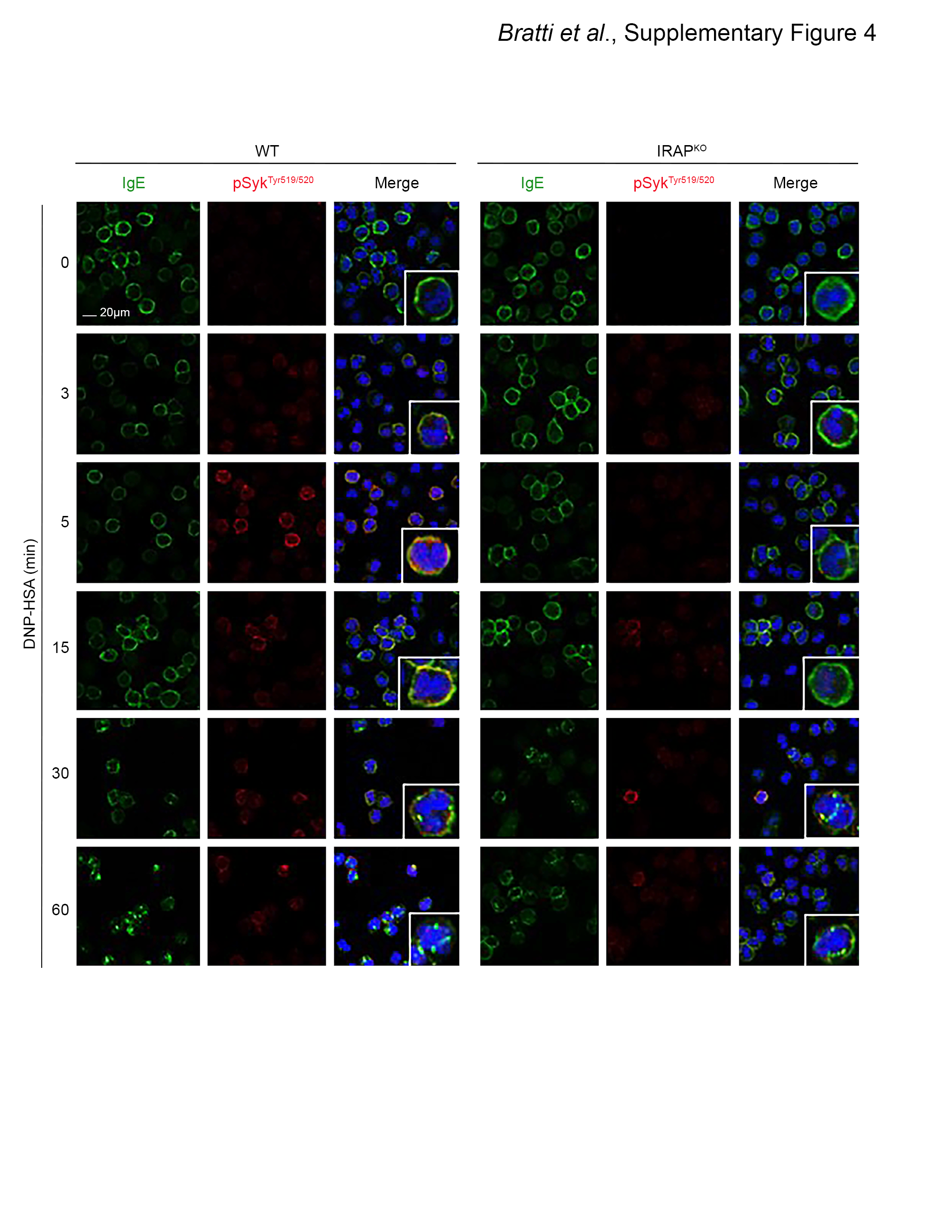
**

**Supplementary Figure 4:**  Kinetic analysis of pSyk staining in WT and IRAP^KO^ BMMCs. Wild-type (WT) and IRAP-deficient (IRAP^KO^) BMMCs were sensitized with anti-DNP IgE for 24 hours and were then plated on fibronectin-coated glass coverslips. Cells were then stimulated with DNP-HSA (30 ng/mL) for indicated time points. Cells were then fixed, permeabilized, and stained with DAPI (blue), anti-IgE (green) and anti-pSyk^Y529/520^ (red) as indicated. Cells were analyzed by confocal microscopy. Images show representative sections (out of 3 experiments) with multiple cells for IgE and pSyk single staining as well as the merge of all colors.

**
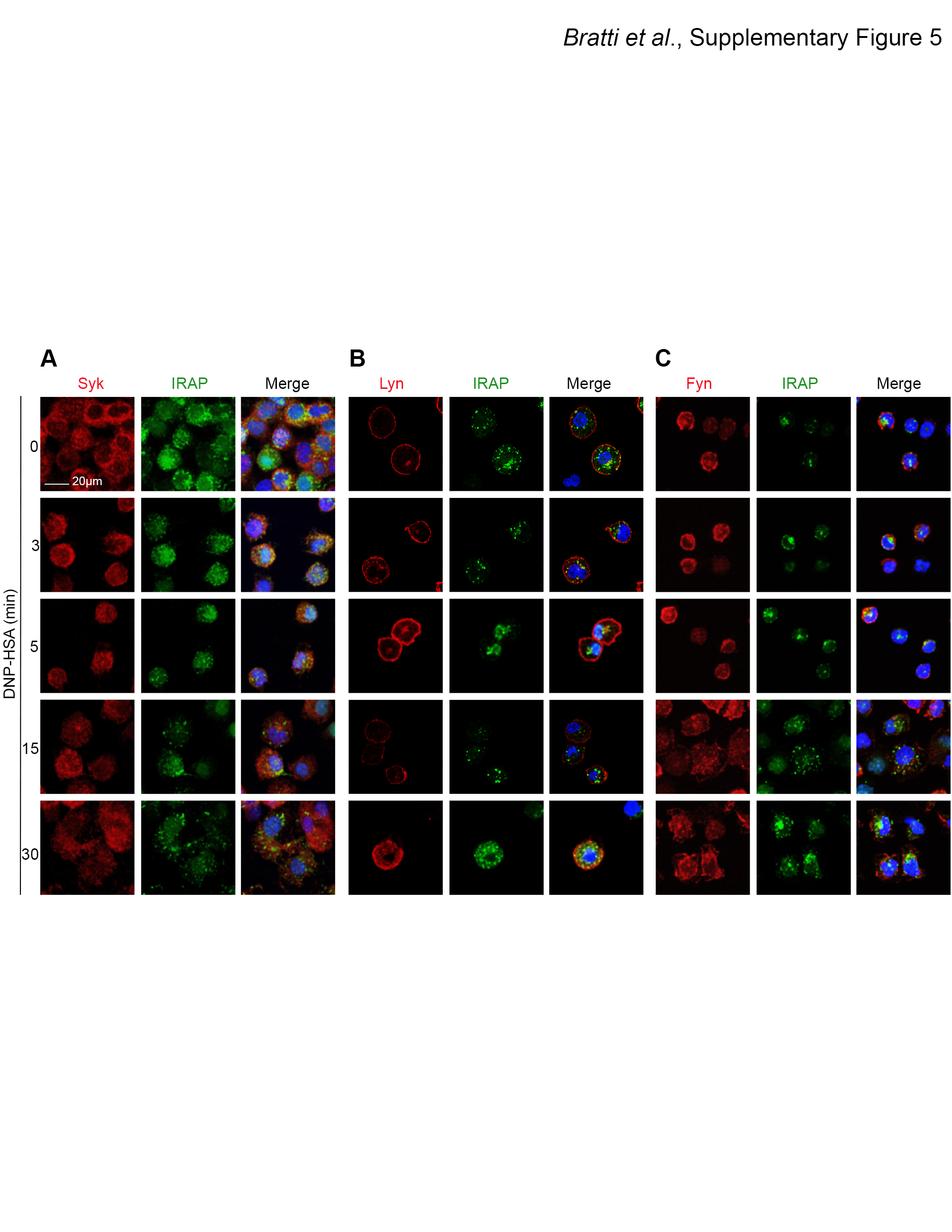
**

**Supplementary Figure 5:**  No colocalization between Syk, Lyn or Fyn and IRAP in FcεRI stimulated BMMCs. WT BMMCs were sensitized with anti-DNP IgE for 24 hours and were then plated on fibronectin-coated glass coverslips. Cells were then stimulated with DNP-HSA (30 ng/mL) for indicated time points. After fixation and permeabilization cells were stained with DAPI (blue), anti-IRAP (green) and anti-Syk **(A)**, anti-Lyn **(B)** or anti-Fyn **(C)** (red) Abs as indicated. Cells were analyzed by confocal microscopy. Images show representative sections (out of 2 experiments) with multiple cells of the indicated single stainings as well as the merge of all colors.
